# Experimental evidence reveals the mobilization and mineralization processes of rare earth elements in carbonatites

**DOI:** 10.1126/sciadv.adm9118

**Published:** 2024-07-03

**Authors:** Xueyin Yuan, Zhiming Yang, Robert A. Mayanovic, Zengqian Hou

**Affiliations:** ^1^ SinoProbe Lab, Institute of Mineral Resources, Chinese Academy of Geological Sciences, Beijing 100037, China.; ^2^ SinoProbe Lab, Institute of Geology, Chinese Academy of Geological Sciences, Beijing 100037, China.; ^3^ Department of Physics, Astronomy and Material Sciences, Missouri State University, Springfield, MO 65897, USA.

## Abstract

Whereas the genesis of carbonatitic rare earth element (REE) deposits has long been a focus of study, the controls on mobilization and mineralization of REEs during magmatic-hydrothermal processes still remain open to debate. Here, we present our investigation of the dissolution and crystallization of REE (fluor)carbonate minerals in alkaline carbonate brine-melts up to 850°C and 11.6 kbar. Our results show that REEs are soluble in Na_2_CO_3_ brine-melts, achieving concentrations exceeding 8 weight % at temperatures above 650°C. The addition of calcium and/or fluoride has minimal impact on REE mobilization, whereas introduction of silica suppresses REE solubilities by half, due to britholite formation above 550°C. Upon cooling, sodium and REEs combine to crystallize in burbankite or carbocernaite in sodium-enriched brine-melts, even at fluoride saturation. However, while the brine-melts contain substantial ferro- or aluminosilicate, REE mineralization in fluorcarbonates occurs after sufficient sodium precipitation in alkaline silicate minerals, hence revealing how silicate and sodium carbonate govern REE mineralization.

## Teaser

Na, Si, Fe, and Al play a vital role in the mobilization and mineralization of REEs in carbonatites.

## INTRODUCTION

The rare earth elements (REEs; Y and the lanthanides La-Lu) are of strategic importance for renewable energy, catalysis, electronics manufacturing, and modern defense systems (*1*, *2*). Globally, carbonatites host over half of the REE resources and account for the majority of REE current production (*2*, *3*). The metallogeny of REEs in carbonatites has been extensively investigated, wherein studies have primarily focused on enrichment and mineralization of the REEs during magmatic and post-magmatic hydrothermal processes. Investigations of melt-fluid inclusions (*1*, *4*–*6*) and high pressure-temperature (*P-T*) studies (*7*–*9*) show that the fractional crystallization of calcite and dolomite results in the residual carbonatitic magmas being progressively enriched in volatiles, alkalis, and incompatible trace elements, including the REEs. In some deposits (e.g., Mountain Pass), the enrichment of REEs during magmatic evolution resulted in mineralization of REE fluorcarbonates having modal abundances at ore-grade concentrations (*2*, *10*). Nevertheless, there is also sufficient evidence to support the transport and mineralization of REEs in hydrothermal fluids, resulting in the development of other giant carbonatite REE deposits (e.g., Maoniuping). This includes fluid inclusion microthermometry data indicating relatively low *P-T* conditions (240° to 500°C and <1 kbar) during REE mineralization (*2*, *11*–*15*) and experimental evidence for substantial REE mobility in aqueous fluids containing halogen, carbonate, and sulfate ions (*16*–*23*). However, mass transport and pronounced enrichment of REEs in hydrothermal fluids are questionable, as fluids clearly exsolved from carbonatitic magmas under shallow crustal conditions are dilute [salinity < 20 weight % or (wt %)] and barren (*24*–*26*), with REEs preferentially partitioning into carbonatitic magmas rather than synmagmatic fluids ($D_{\text{REE}}^{F/M}$ = 0.02 to 0.15) (*27*). Our recent study of the melt-fluid transition in alkaline carbonatitic systems (*28*) revealed that, under *P-T* conditions (e.g., >600°C and 3.0 kbar) corresponding to the magmatic-hydrothermal transition in deep-seated intrusions (*6*, *29*–*31*), the evolution from carbonatitic magmas to hydrothermal fluids is continuous without the occurrence of melt-fluid immiscibility. Specifically, the condensed hypersaline REE ore-forming liquids (*30*, *32*–*34*) are in reality highly evolved carbonatitic melts, or, as commonly referred to, carbonate brine-melts (*28*, *35*, *36*), rather than hydrothermal fluids exsolved from the melts. To better understand the enrichment and mineralization of REEs during continuous melt-fluid evolution under deep crustal conditions, variations in REE solubilities and *P-T* conditions for crystallization of REE (fluor)carbonate minerals during cooling and decompression of carbonate brine-melts need to be better constrained.

A comprehensive description of the nature of complexes responsible for REE transport in carbonate brine-melts and/or associated hydrothermal fluids, resulting in the development of carbonatitic REE deposits, is still lacking. Molecular dynamics simulations (*37*) indicate that the complexation between REEs and carbonate ions is orders of magnitude more stable than that of other species involving sulfate, halogen, or nitrate ions. However, given the widespread occurrence of REEs as fluorcarbonates (bastnäsite, parisite, synchysite, etc.) in carbonatites, fluoride, and carbonate ions are generally regarded as major precipitation agents (*17*, *19*). The extensive occurrence of bastnäsite with baryte (*2*, *10*, *31*, *38*) and the abundance of sulfate daughter minerals (thénardite, arcanite, celestine, etc.) in melt-fluid inclusions from carbonatites (*11*–*13*, *30*, *31*, *33*) indicate that REE mobilization may be governed by complexation with sulfate ions. This is further supported by the results from hydrothermal experiments, where REE-rich sulfate liquids were observed to form via liquid-melt or liquid-liquid immiscibility in (Na-)REE-SO_4_ hydrothermal systems (*16*, *18*). Nevertheless, the roles of sulfate and carbonate ions in REE mobilization need to be reevaluated thoroughly, as the occurrence of melt-fluid inclusions enriched in REEs but depleted in sulfate ions (*30*, *34*, *39*) indicates that substantial REE solubilities can be achieved without REE-SO_4_^2−^ complexing. More notably, REEs in carbonatites have been shown to be highly mobile under the presence of alkalis during high *P-T* experiments (*7*). Here, we present the results from our investigations of REE solubilities in high *P-T* Na_2_CO_3_ brine-melts that clearly delineate the fundamental roles of sodium, silica, and carbonate components during REE mobilization. Furthermore, by comparing the mineralization of REEs as alkaline carbonate and/or fluorcarbonate minerals from carbonatitic melts with compositions varying from haplo Na_2_CO_3_ brine-melts to those enriched in calcium, fluoride, silica, iron or aluminum, we provide unambiguous evidence for delineating the precise nature of the REE ore-forming processes in carbonatites.

## RESULTS

### Dissolution of carbocernaite and bastnäsite in Na_2_CO_3_ brine-melts

**Fig. 1.**
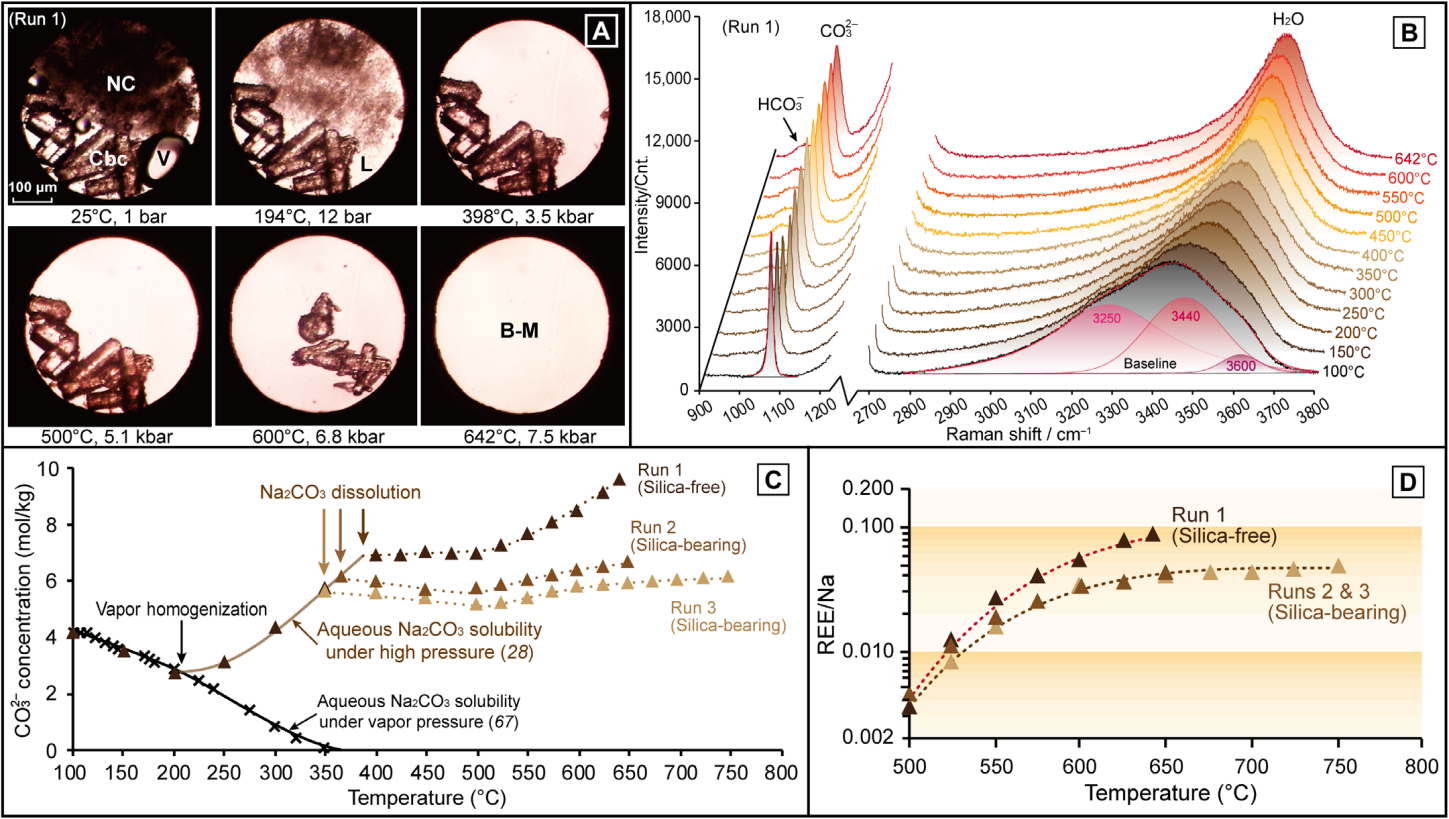
Dissolution of carbocernaite in Na_2_CO_3_ brine-melt. (**A**) Photomicrographs showing the dissolution of synthetic carbocernaite (Cbc) in Na_2_CO_3_ brine-melt (run 1). NC, L, V, and B-M are for Na_2_CO_3_·nH_2_O, aqueous liquid, vapor bubble, and Na_2_CO_3_ brine-melt, respectively. (**B**) In situ Raman spectra showing the variation in CO_3_^2−^ Raman peak intensity under high *P-T* conditions (run 1). Cnt., counts. (**C**) Variations in CO_3_^2−^ concentration during the dissolution of natrite (below 400°C) and carbocernaite (above 500°C); Na_2_CO_3_ solubility under vapor pressure (same in Fig. 2C) is from (*67*). (**D**) Increase in REE/Na molar ratio as a function of temperature in the absence (run 1) and presence (runs 2 and 3) of silica.

**Fig. 2.**
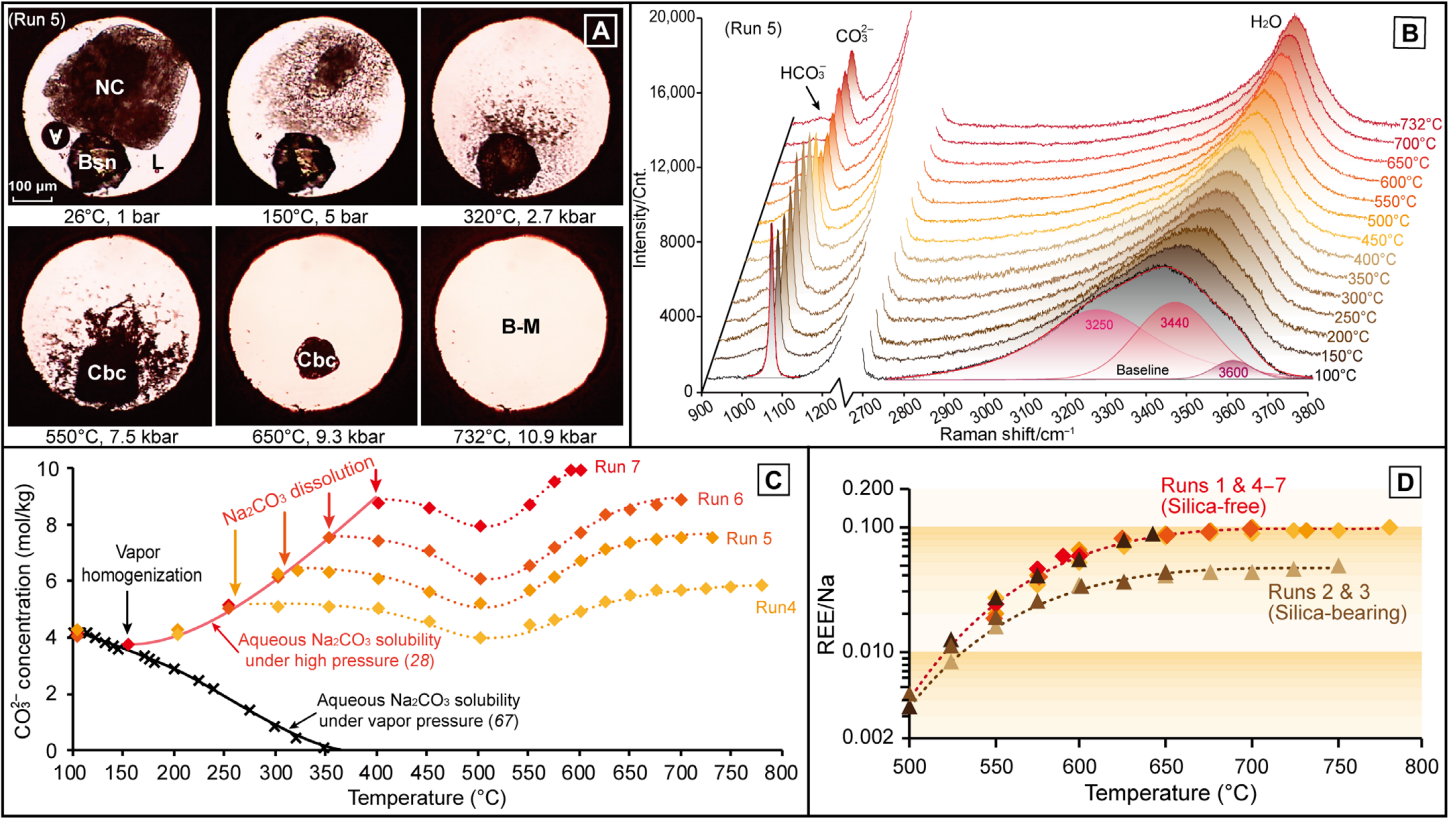
Dissolution of bastnäsite in Na_2_CO_3_ brine-melt. (**A**) Photomicrographs showing the transformation from bastnäsite (Bsn) to carbocernaite (Cbc) and the dissolution of carbocernaite in Na_2_CO_3_ brine-melt (run 5). Abbreviations are the same as those in Fig. 1. (**B**) In situ Raman spectra showing the variation in CO_3_^2−^ Raman peak intensity under high *P-T* conditions (run 5). (**C**) Variations in CO_3_^2−^ concentration, in which the decrease in CO_3_^2−^ concentration between 300° and 500°C was due to reaction between bastnäsite and Na_2_CO_3_, and the subsequent increase in CO_3_^2−^ concentration above 500°C was caused by dissolution of carbocernaite. (**D**) Increase in REE/Na molar ratio from runs 1 and 4 to 7 as a function of temperature above 500°C and those from silica-bearing runs 2 and 3 are shown for comparison.

Table 1 lists the mineral assemblages, the *P-T* conditions, and the end products of the experimental runs executed in this study. Figures 1 and 2 show the in situ observational and Raman spectral data acquired from REE dissolution experiments using synthetic carbocernaite [Cbc; NaCe(CO_3_)_2_] and natural bastnäsite [Bsn; (Ce,La)CO_3_F] in Na_2_CO_3_ brine-melts, respectively. Variations in CO_3_^2−^ concentration and REE/Na molar ratio in the brine-melts as a function of temperature, as determined from multiple runs (1 to 7) using a Raman quantification method outlined in our previous study (*28*), are shown for carbocernaite and bastnäsite dissolution experiments in Figs. 1 and 2 (C and D), respectively. Because of the prograde Na_2_CO_3_ solubility in high density (>1.0 g/cm^3^) aqueous liquids (*28*), natrite (Na_2_CO_3_) dissolved between 270° and 398°C (Figs. 1A and 2A). The Na_2_CO_3_ concentrations in this study, ranging from 5.20 to 8.92 mol/kg (35.5 to 48.6 wt %; table S1), exceeded the upper critical point (~5.0 mol/kg or 35 wt %) of the Na_2_CO_3_-H_2_O system (*40*), thus indicating that the aqueous Na_2_CO_3_ liquids studied were condensed brine-melts (*28*). As shown in Fig. 1C, the CO_3_^2−^ concentration in the silica-free run 1 remained fixed at ~7.0 mol/kg up to 500°C, upon dissolution of natrite at 398°C and 3.5 kbar, indicating that dissolution of carbocernaite below 500°C is below the detection limit by using the Raman quantification method. Thereafter, the CO_3_^2−^ concentration in the brine-melt increased monotonically upon progressive dissolution of carbocernaite and reached 9.62 mol/kg at 642°C and 7.5 kbar (table S1 and Fig. 1C). Given the Ce:Na:CO_3_^2−^ molar ratio of 1:1:2 in carbocernaite, the additional increase in CO_3_^2−^ concentration by 2.69 mol/kg corresponds to a cerium (Ce) mass concentration of 8.6 wt % (Table 1) in the brine-melt.

**Table 1.** Summary of the mineral assemblages, the *P-T* conditions, and the end products from runs 1 to 11, S1, and S2. Aeg, aegirine; Bri, britholite; Bsn, bastnäsite; Cal, calcite; Can, cancrinite; Cbbn, calcioburbankite; Cbc, carbocernaite; Crn, corundum; Cry, cryolite; Flr, fluorite; Hem, hematite; NC, Na_2_CO_3_·nH_2_O; Nye, nyerereite; Qz, quartz; Vll, villiaumite.

Run	Starting materials	*P-T* condition*	Homogenized brine-melt composition†			Crystallization products‡
			Na_2_O wt %	REE wt %	(REE/Na)_mol_	
1	Cbc + NC + H_2_O	642°C, 7.5 kbar	17.5	8.6	0.089	Cbc, NC
2	Cbc + NC + Qz + H_2_O	650°C, 8.0 kbar	16.9	3.8	0.042	Bri, Qz, Cbc, NC
3	Cbc + NC + Qz + H_2_O	750°C, 9.9 kbar	14.3	4.0	0.047	Bri, Qz, Cbc, NC
4	Bsn + NC + H_2_O	780°C, 11.6 kbar	13.9	6.4	0.093	Cbc, Vll
5	Bsn + NC + H_2_O	732°C, 10.9 kbar	15.4	8.3	0.091	Cbc, Vll, NC
6	Bsn + NC + H_2_O	700°C, 10.1 kbar	16.8	8.7	0.092	Cbc, Vll, NC
7	Bsn + NC + H_2_O	595°C, 8.8 kbar	18.7	7.2	0.055	Cbc, Vll, NC
8	Bsn + NC + Cal + H_2_O	690°C, 9.5 kbar	–	–	–	Cal, Cbbn, Nye, Flr, NC
9	Bsn + NC + Flr + Qz + Hem+H_2_O	850°C, 11.4 kbar	–	–	–	Bri, Flr, Aeg, Bsn, Qz, Cbc, NC
10	Bsn + NC + Flr + Qz +Hem+H_2_O	820°C, 11.8 kbar	–	–	–	Bri, Flr, Aeg, Bsn, Cbbn, NC
11	Bsn + NC + Flr + Qz +Crn + H_2_O	765°C, 9.0 kbar	–	–	–	Bri, Can, Bsn, Nye, Flr, Cbbn, Cry
S1	Bsn + NC + H_2_O	680°C, 4.5 kbar	–	–	–	Cbc, Vll
S2	Bsn + NC +Cal + H_2_O	675°C, 3.8 kbar	–	–	–	Cbbn, Nye, Vll, NC

*Pressure was estimated using the isochoric *P-T* curves defined for the NaCl-H_2_O system (runs 1, 4 to 7, and S1) or frequency shifts in the Raman peak of quartz at 464 cm^−1^ (runs 2, 3, and 9 to 11) or the peak of calcite at 1086 cm^−1^ (runs 8 and S2).

†Brine-melt compositions from runs 8 to 11, S1, and S2 were not determined, as CO_3_^2−^ concentration in these runs was affected by different processes, including dissolutions of carbocernaite, nyerereite, or calcite; interaction with quartz or other silicates; and exsolution of aqueous fluids.

‡Minerals were listed according to the crystallization sequence during cooling, calcite in run 8 transformed into nyerereite around 610°C, and britholite in runs 9 to 11 transformed partially or completely into bastnäsite below 600°C.

**Fig. 3.**
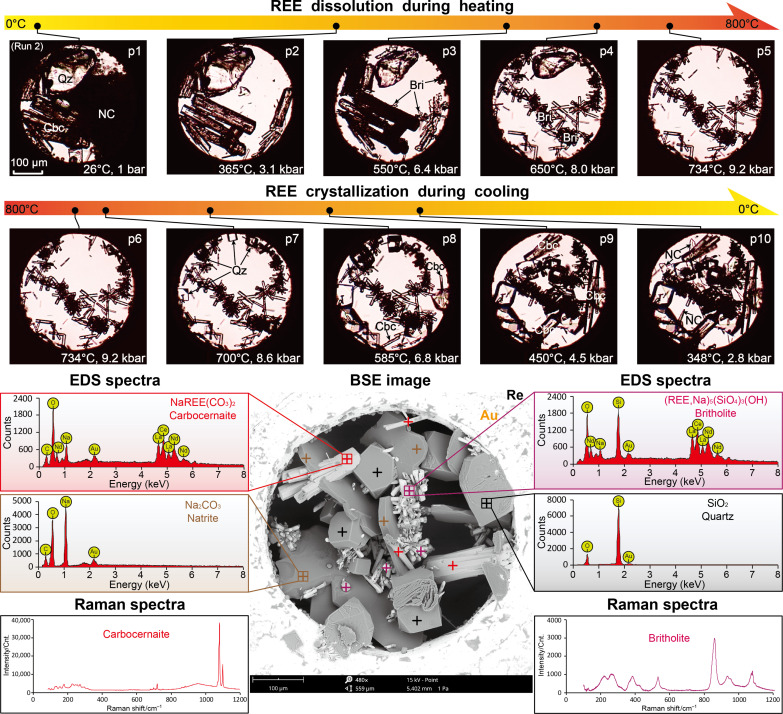
Dissolution and crystallization of REEs in silica-saturated Na_2_CO_3_ brine-melt (run 2). Photomicrographs p1 to p5 and p6 to p10 show the phase changes observed during heating and cooling, respectively. Britholite (Bri) formed from reaction between carbocernaite (Cbc) and quartz (Qz) above 550°C during heating and remained stable thereafter. A back scattered electron (BSE) image of the end product, together with energy-dispersive x-ray spectrometer (EDS) and Raman spectra of britholite, carbocernaite, natrite (NC), and quartz, is shown for identification of the REE mineralization products.

Adding quartz (Qz; SiO_2_) to the system in runs 2 and 3 reduced the CO_3_^2−^ concentration in the brine-melts by about 0.4 mol/kg between 350° and 500°C (table S1 and Fig. 1C), through partial transformation of CO_3_^2−^ ions to HCO_3_^−^ and CO_2_ (*41*). At temperatures (*T*) > 550°C, interaction between carbocernaite and quartz suppressed REE solubilities by forming hexagonal britholite [Bri; (Ca,Na,REE)_5_(SiO_4_)_3_(OH)] clusters (Fig. 3, p3 to p5): 4NaREE(CO_3_)_2_ + 3SiO_2_ + 2H_2_O → (Na,REE)_5_(SiO_4_)_3_(OH) + 3Na^+^ + 3HCO_3_^−^ + 5CO_2_. This reaction released HCO_3_^−^ and CO_2_ (both were detected by using the Raman spectroscopy, after the sample was cooled down to room temperature) but had negligible influence on CO_3_^2−^ concentration in the brine-melt. Consequently, the increase in CO_3_^2−^ concentration by 0.98 and 0.96 mol/kg in runs 2 and 3, at temperatures ranging from 500° to 650°C and 500° to 750°C, respectively (table S1 and Fig. 1C), was dominated by dissolution of carbocernaite. The REE mass concentrations in the brine-melts from runs 2 and 3 were estimated to be 3.8 and 4.0 wt %, respectively, approximately half of that in run 1 (Table 1). Nevertheless, these values agree well with the bulk compositions of melt-fluid inclusions (Na_2_CO_3_, 36 wt %; REEs, 3 wt %) from carbonatites (*34*, *39*) and are fully consistent with natural ore-forming liquids being undersaturated with REEs.

Before dissolution into the Na_2_CO_3_ brine-melt, the bastnäsite fragments used in runs 3 to 7 first transformed slowly into carbocernaite via reaction with Na^+^ and CO_3_^2−^ above 200°C (Fig. 2A): Na^+^ + CO_3_^2−^ + (Ce,La)CO_3_F → Na(Ce,La)(CO_3_)_2_ + F^−^. As confirmed from our separate experiments that are not reported here, this reaction is crucial for REE dissolution. In these experiments that were made with other aqueous salt solutions (K_2_CO_3_, Na_2_SO_4_, NaCl, etc.), we found no evidence for transformation into carbocernaite or appreciable dissolution of bastnäsite under similar high *P-T* conditions. The bastnäsite-to-carbocernaite reaction proceeded at an increasing rate with increasing temperature, until completion while the samples were held at 500°C for 1 to 3 hours, as monitored by the disappearance of the bastnäsite Raman peaks (fig. S1). As shown in table S1 and Fig. 2C, a pronounced minimum occurs around 500°C, in the trends of the CO_3_^2−^ concentration in the brine-melts, with minimum values ranging from 4.11 to 8.07 mol/kg. Beyond the minimum and in conjunction with carbocernaite dissolving into the brine-melts upon heating to higher temperatures (Fig. 2A), the CO_3_^2−^ concentration increased further and then plateaued at values in the range of 5.99 to 10.06 mol/kg, under *P-T* conditions between 595°C and 8.8 kbar and 780°C and 11.6 kbar (table S1 and Fig. 2C). Therefore, in comparison to the initial Na_2_CO_3_ concentration of 5.20 to 8.92 mol/kg (35.5 to 48.6 wt %) in the brine-melts, the homogenization of bastnäsite (via the transformation into carbocernaite) resulted in additional increase in CO_3_^2−^ concentration by 0.79 to 1.31 mol/kg. This corresponds well to the REE mass concentrations (calculated using the average atomic weights of La and Ce) ranging between 6.4 and 8.7 wt % (Table 1).

In direct reflection of carbocernaite dissolution at *T* > 500°C, the REE/Na molar ratios in runs 1 to 7, as calculated from variations in CO_3_^2−^ concentration in the brine-melt, are shown in table S1 and plotted in Figs. 1D and 2D. It can be seen that the REE/Na molar ratio in runs 1 and 4 to 7 increased rapidly upon heating from below 0.005 at 500°C to 0.09 at 650°C and then reached steady-state values between 0.09 and 0.10 at temperatures above 650°C. This indicates that REE solubilities in Na_2_CO_3_ brine-melts increased directly with increasing Na_2_CO_3_ molarity, with no detectable influence from the addition of fluoride or variations in pressure over a wide range. The REE/Na molar ratio in silica-bearing runs 2 and 3 also increased, but at a much lower rate from below 0.005 at 500°C to 0.05 at 750°C, thus confirming that the presence of silica inhibits REE mobilization in alkaline carbonatitic brine-melts (*7*, *42*). By fitting the logarithm of REE/Na molar ratios as a function of temperature, it can be further inferred that REE solubilities in the brine-melts decreased from several weight percent at 650°C to tens of parts per million (ppm) at 400°C. The estimated REE solubilities, although with large uncertainties, are roughly consistent with the increasing lanthanum (La) solubility in aqueous Na_2_CO_3_ solutions from <10 to hundreds of ppm in the temperature range of 300° to 500°C (*20*), which indicates that REEs are highly immobile during post-magmatic hydrothermal activity.

### Crystallization of carbocernaite and burbankite from Na_2_CO_3_ brine-melts

Whereas the phase changes observed during heating and cooling in run 1 were reversible, the resulting mineral assemblages upon cooling of the REE-rich Na_2_CO_3_ brine-melts in runs 2 to 7 were distinct from the ones initially loaded into the HDAC. For instance, britholite as formed via interaction between carbocernaite and quartz at *T* > 550°C remained stable during the subsequent heating and cooling processes in silica-bearing runs 2 and 3 (Fig. 3). For runs 4 to 7 with bastnäsite and natrite as the starting materials, carbocernaite mineralized from the brine-melts at temperatures of 30° to 60°C lower than the homogenization points, then grew at a decreasing rate down to 500° to 460°C, and remained stable at lower temperatures (fig. S1). The incorporation of sodium into carbocernaite either prohibited natrite precipitation (run 4, fig. S1) or lowered the *P-T* conditions for natrite precipitation by tens of degrees (runs 5 to 7). Analyses of the end products, using a scanning electron microscope with an energy-dispersive x-ray spectrometer (SEM-EDS), showed that the mineralization products from runs 4 to 7 included prismatic carbocernaite and euhedral villiaumite (Vll; NaF) crystals (fig. S1; the soluble Na_2_CO_3_ hydrates were removed during sample cleaning; see Materials and Methods). No bastnäsite or REE fluoride (e.g., fluocerite) was detected, implying that fluoride was highly incompatible during REE precipitation from Na_2_CO_3_ brine-melts.

**Fig. 4.**
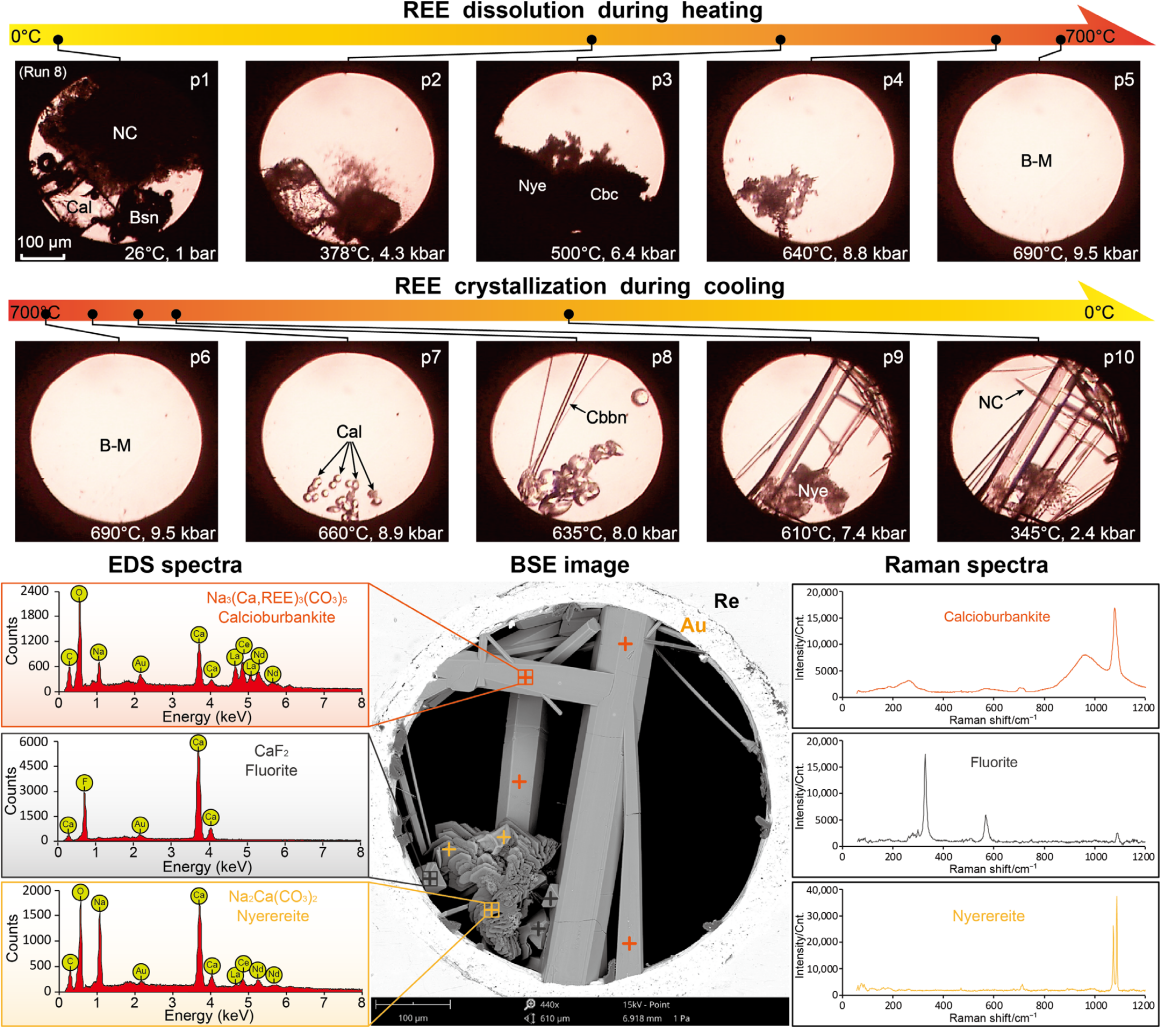
Dissolution and crystallization of REEs in Ca-bearing carbonate brine-melt (run 8). Photomicrographs p1 to p5 show the dissolution of natrite (NC), bastnäsite [Bsn; transforming to carbocernaite (Cbc) above 200°C] and calcite [Cal; transforming to nyerereite (Nye) above 350°C] into alkaline carbonate brine-melt (B-M); p6 to p10 show the crystallization of calcite (transformed to nyerereite below 610°C), calcioburbankite (Cbbn), and natrite during cooling of the brine-melt. BSE image of the end product and representative EDS and Raman spectra of calcioburbankite, nyerereite, and fluorite are shown for identification of the REE mineralization results.

Because highly evolved carbonatitic magmas or brine-melts are saturated with calcite or dolomite (*1*, *8*), calcium behavior during REE mineralization was investigated by incorporating calcite (Cal; CaCO_3_) as a starting material alongside bastnäsite and natrite in run 8 (Fig. 4). Notable phase transformations were observed upon heating, including the bastnäsite-to-carbocernaite and calcite-to-nyerereite [Nye; Na_2_Ca(CO_3_)_2_] transformations above 200° and 350°C (Fig. 4, p2 and p3), respectively. Thereafter, dissolution of carbocernaite and nyerereite occurred above 500°C, and the sample homogenized at 690°C and 9.5 kbar (Fig. 4, p3 to p5). REE concentration in the brine-melt was not determined, because the increase in CO_3_^2−^ concentration as caused by dissolution of carbocernaite and nyerereite could not be differentiated. Nevertheless, we infer from the similar bastnäsite-to-carbocernaite transformation and carbocernaite dissolution processes between run 8 (Fig. 4) and runs 4 to 7 (fig. S1) that the addition of calcite has a limited effect on REE solubilities in Na_2_CO_3_ brine-melts. During cooling of the sample in run 8, calcite and calcioburbankite [Na_3_(Ca,Ce,La)_3_(CO_3_)_5_ crystallized sequentially at 660° and 635°C, respectively (Fig. 4, p7 and p8). The calcite crystals then transformed to nyerereite at ~610°C (Fig. 4, p9), which is consistent with the autometasomatism of calcite by the remaining alkaline melt during the evolution of carbonatitic magmas (*8*, *28*). With further decrease in temperature, the growth in calcioburbankite prisms tapered off around 480°C, and the remaining Na_2_CO_3_ in the brine-melt crystallized into natrite below 345°C (Fig. 4, p10). SEM-EDS analysis showed that, in addition to those in calcioburbankite, minor quantities of REEs were also detected in the nyerereite crystals (Fig. 4), which is consistent with the REE partition coefficients between nyerereite and natrocarbonatite melt being in the range of 0.27 to 0.58 (*43*). Fluoride did not precipitate with REEs in the mineral products but crystallized as fluorite during a later hydrothermal stage (not clearly observed but estimated to occur below 400°C), indicating that REE fluorcarbonate minerals (e.g., bastnäsite) are unlikely to crystallize directly from sodium-enriched carbonatitic melts or brine-melts, even at fluoride saturation.

**Fig. 5.**
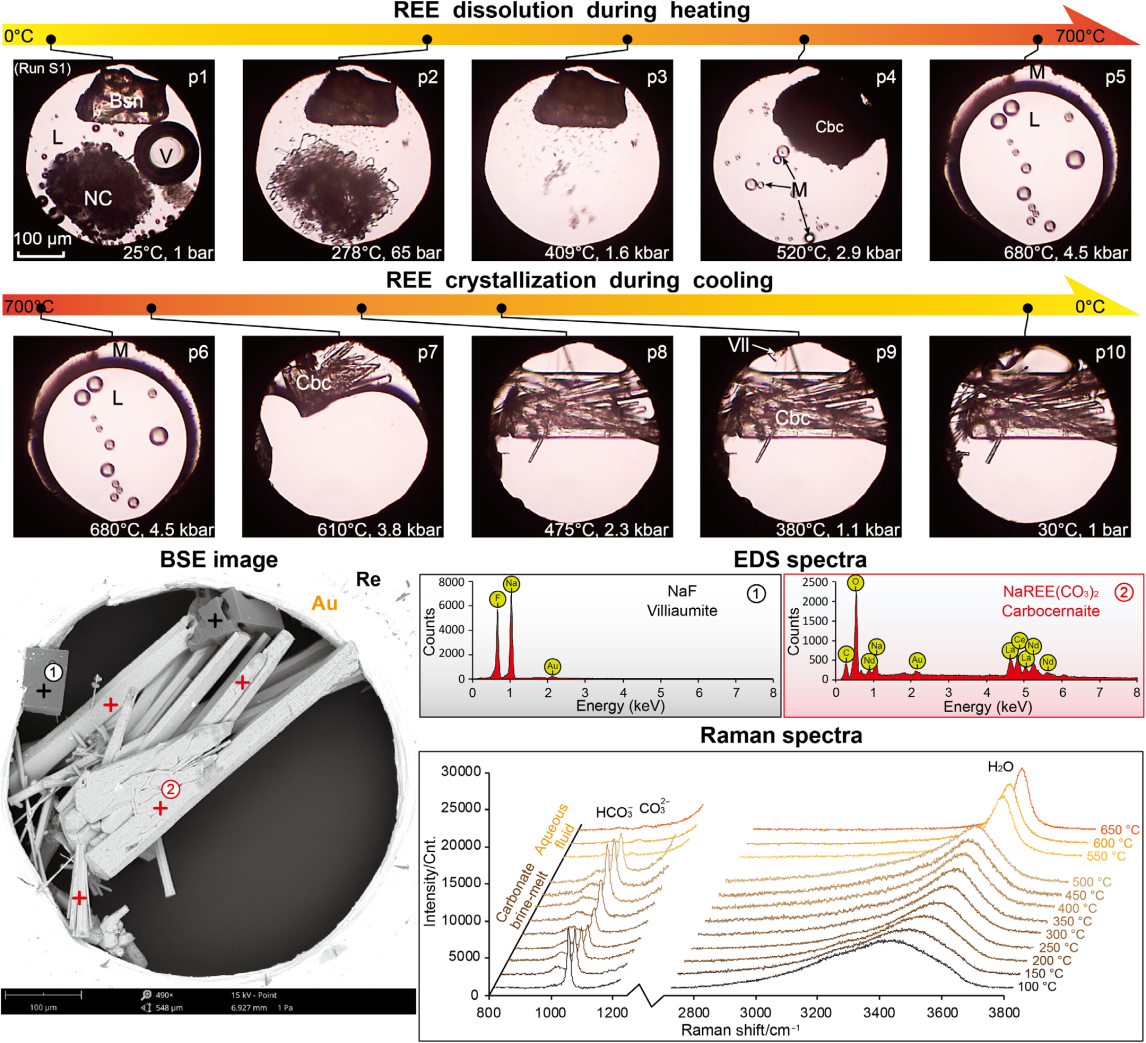
Dissolution and crystallization of REEs during melt-fluid immiscibility. Photomicrographs p1 to p5 show the immiscibility between a carbonate melt (M) and an aqueous fluid (L) above 520°C, with carbocernaite [Cbc; formed from reaction between bastnäsite (Bsn) and natrite (NC) above 200°C] dissolving into the melt at 680°C; p6 to p10 show the crystallization of carbocernaite and villiaumite (Vll) from the melt and aqueous fluid, respectively. BSE image of the end product and EDS spectra of villiaumite and carbocernaite are shown for identification of the REE mineralization results. Raman spectra of carbonate brine-melt (<500°C) and hydrothermal fluid (550° to 650°C) show the variation in liquid composition upon melt-fluid immiscibility.

We also investigated REE deposition during melt-fluid immiscibility under high temperature (>500°C) but relatively low pressure (<4.5 kbar) conditions (see in the Supplementary Materials). During these investigations, carbocernaite and calcioburbankite were observed to crystallize from the carbonate melt phase between 650° and 480°C, in the absence (run S1) or the presence (run S2) of calcite, respectively (Fig. 5 and fig. S2). In addition, Raman spectra measured from the fluid phase revealed that the aqueous fluids were dilute, with CO_3_^2−^ and HCO_3_^−^ Raman bands being barely perceptible (Fig. 5). The CO_3_^2−^ concentration was estimated to be ~2.0 mol/kg or 18 wt %, which is consistent with the exsolution of dilute and barren hydrothermal fluids from alkaline carbonatite melts (*28*). These results, along with REEs partitioning preferentially into carbonatitic melts over hydrothermal fluids (*27*), demonstrate clearly that, upon melt-fluid immiscibility under shallow crustal conditions or during brecciation of the surrounding rocks (*30*, *35*, *44*), enrichment and deposition of REEs are limited within the alkaline carbonate melts, irrespective of the melt being potentially dry and enriched in CO_2_ (*45*): We view this as further evidence for the mineralization of REE carbonate minerals during a late magmatic stage (*46*–*48*). Once again, fluoride in both samples remained incompatible during REE deposition and precipitated as villiaumite from the hydrothermal fluid at lower temperatures (<400°C; Fig. 5).

### Mineralization of REEs in bastnäsite from Si-Fe– or Si-Al–bearing carbonate brine-melts

**Fig. 6.**
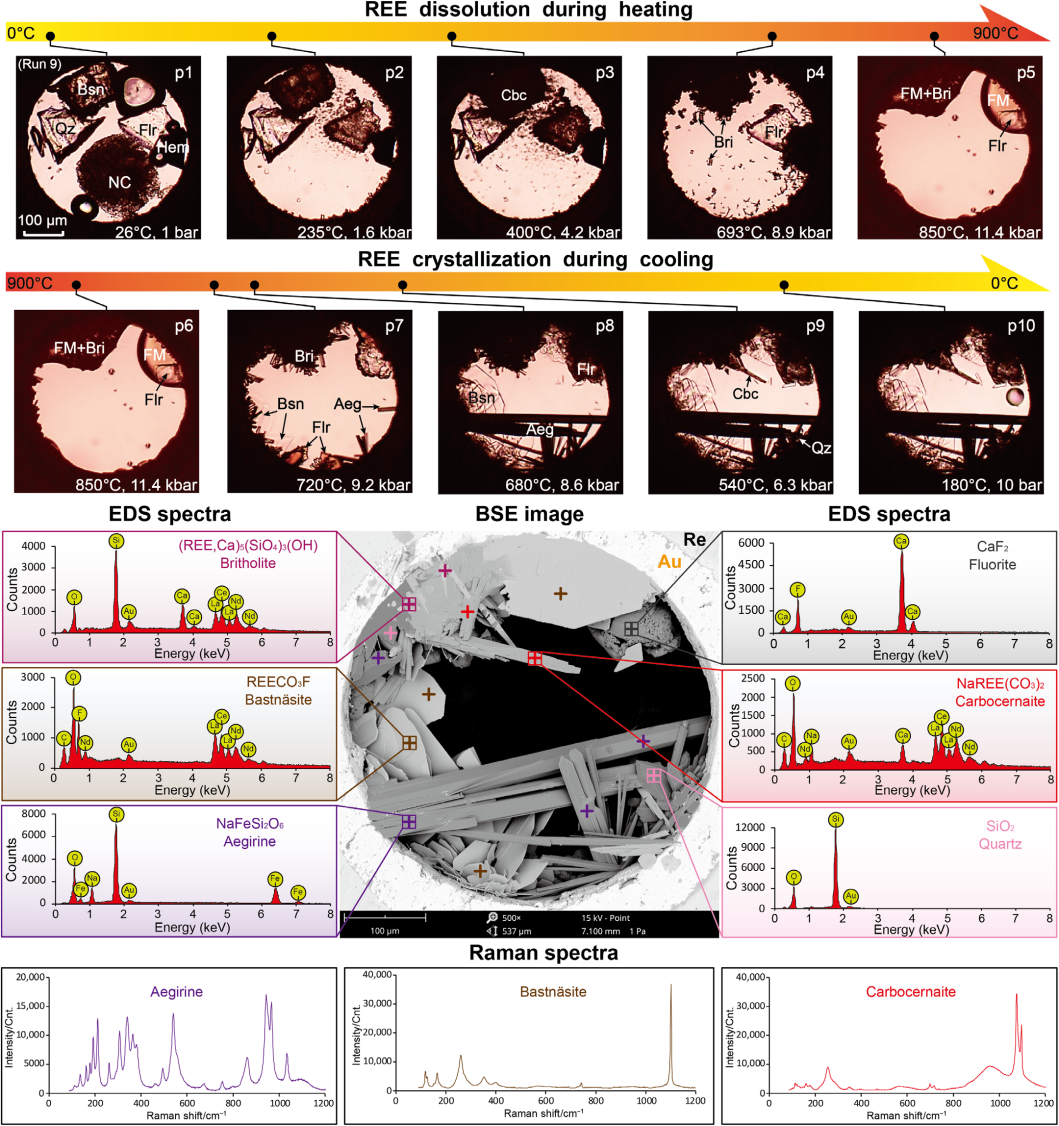
Dissolution and crystallization of REEs in Si-Fe–bearing carbonate brine-melt (run 9). Photomicrographs p1 to p5 show the dissolution of natrite (NC), bastnäsite (Bsn), quartz (Qz), and hematite (Hem) into a carbonate brine-melt, the formation of britholite (Bri) above 550°C, and melting of the remaining fluorite (Flr) into a fluoride melt (FM); p6 to p10 show the sequential crystallization of fluorite, aegirine (Aeg), bastnäsite, and carbocernaite (Cbc) during cooling of the sample. BSE image of the end product, together with EDS and Raman spectra of britholite, carbocernaite, bastnäsite, aegirine, fluorite, carbocernaite, and quartz are shown for identification of the mineralization products.

The emplacement of carbonatitic magmas is extensively accompanied by contact metasomatism with silicate wall rocks, leading to substantial changes in magma compositions through loss of alkalis and assimilation of ferro- and/or aluminosilicates (*2*, *31*, *35*, *38*, *44*, *49*). Three additional runs were made to investigate the behavior of REEs in Si-Fe– or Si-Al–bearing carbonatitic melts. The starting mineral assemblages for these runs included bastnäsite, natrite, fluorite, quartz, and hematite (Hem; Fe_2_O_3_; runs 9 and 10) or corundum (Crn; Al_2_O_3_; run 11). The phase changes observed (excluding vapor homogenization at 120° and 92°C in runs 9 and 10, respectively) during heating of the Si-Fe–bearing runs (9 and 10) include natrite dissolution (178° and 316°C), bastnäsite-to-carbocernaite transformation (200° to 500°C, same as those in runs 4 to 8), dissolution of quartz (693° and 550°C), formation of britholite (above 550°C), and, lastly, dissolution of hematite (having partially transformed into aegirine) and melting of the remaining fluorite above 800°C (Fig. 6, p1 to p5, and fig. S3, p1 to p5). These changes resulted in the coexistence among a carbonatitic melt, a fluoride melt (FM) and britholite crystals above 800°C (Fig. 6, p5, and fig. S3, p5). The subsequent cooling process in run 9 can be divided into four stages: (i) crystallization of fluorite from the FM above 720°C (Fig. 6, p7); (ii) mineralization of hexagonal bastnäsite flakes and aegirine (Aeg; NaFeSi_2_O_6_) prisms from the carbonatitic brine-melt, occurring in association with dissolution of britholite from 720° to roughly 600°C (Fig. 6, p7 and p8); (iii) precipitation of carbocernaite and quartz from the remaining brine-melt at temperatures down to 500°C (Fig. 6, p9); and (iv) no minerals were observed to crystallize below 500°C (Fig. 6, p10), implying that the remaining liquid was a barren and dilute hydrothermal fluid that contributed negligibly to REE mineralization. The crystallization in run 10 (fig. S3, p6 to p10) proceeded in a similar manner to that in run 9, except for the precipitation of calcioburbankite instead of carbocernaite and the absence of quartz precipitation during stage (iii). SEM-EDS and Raman analyses showed that the dominant mineralization products from runs 9 and 10 were fluorite, aegirine, bastnäsite, calcioburbankite or carbocernaite, and quartz, which is consistent with the aegirine-augite-fluorite-bastnäsite-quartz mineral paragenesis in the Maoniuping and other carbonatitic REE deposits (*2*, *31*, *38*, *50*, *51*). In particular, the crystallization of carbocernaite or calcioburbankite during stage (iii) indicates that the alkalis in our experiments were oversaturated relative to silica and iron. We infer from this that sufficient precipitation of sodium in aegirine is essential for the subsequent mineralization of REEs as fluorcarbonates rather than burbankite or carbocernaite.

**Fig. 7.**
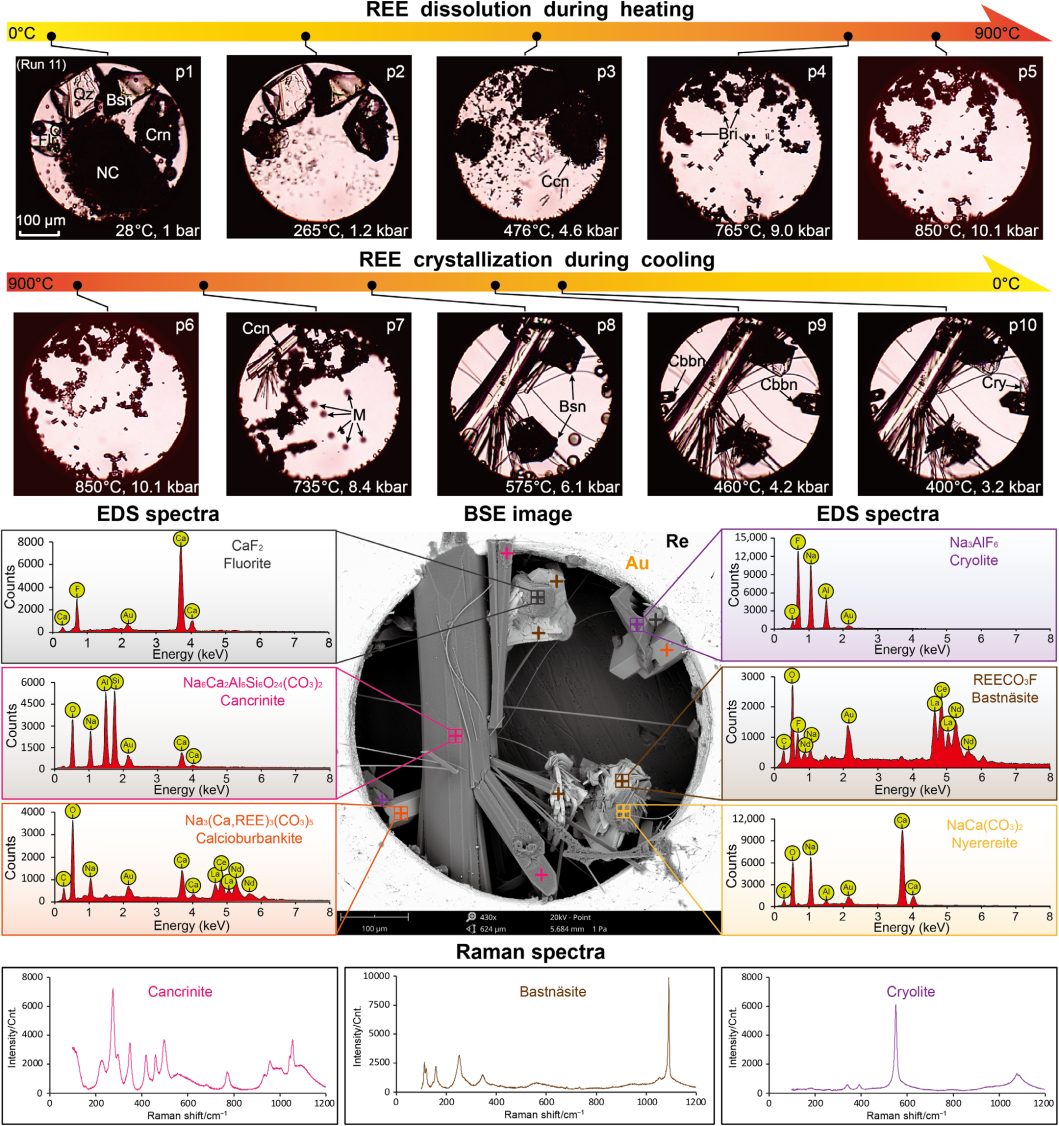
Dissolution and crystallization of REEs in Si-Al–bearing carbonate brine-melt (run 11) Photomicrographs p1 to p5 show the dissolution of natrite (NC), bastnäsite (Bsn), quartz (Qz), fluorite (Flr), and corundum (Crn) into the brine-melt; p6 to p10 show the crystallization of cancrinite (Ccn), bastnäsite, calcioburbankite (Cbbn), and cryolite (Cry) during cooling of the sample. Britholite (Bri) formed above 550°C upon heating, and transformed into bastnäsite below 600°C during cooling. The emergence of melt droplets (M) between 750° and 460°C was probably due to silicate-carbonate melt immiscibility. BSE image of the end product, and EDS and Raman spectra of fluorite, cancrinite, bastnäsite, calcioburbankite, cryolite, and nyerereite are shown for identification of the mineralization products.

Upon heating, the bastnäsite-to-carbocernaite transformation in the Si-Al–bearing run 11 (vapor homogenization and natrite dissolution at 182° and 265°C, respectively) began at a notably higher temperature (280° to 300°C; Fig. 7, p2) than other runs containing bastnäsite in the starting assemblage. Thereafter, the formation of cancrinite [Ccn; Na_6_Ca_2_Al_6_Si_6_O_24_(CO_3_)_2_] via reaction among quartz, fluorite, corundum, and Na_2_CO_3_ brine-melts above 350°C: 6SiO_2_ + 2CaF_2_ + 3Al_2_O_3_ + 6Na^+^ + 8CO_3_^2−^ + 3H_2_O → Na_6_Ca_2_Al_6_Si_6_O_24_(CO_3_)_2_ + 4F^−^ + 6HCO_3_^−^, resulted in complete consumption of quartz at 476°C (Fig. 7, p3). With steady increase in temperature, britholite formed above 550°C, and the remaining carbocernaite, fluorite, and cancrinite dissolved progressively into the brine-melt between 680° and 765°C (Fig. 7, p4). The sample was further heated to 850°C, and no britholite dissolution or other phase transformations were observed (Fig. 7, p5). Crystallization of cancrinite was observed upon cooling at ~740°C, whereupon tiny melt droplets emerged from the brine-melt (Fig. 7, p7). We speculate that emergence of melt droplets (whose composition could not be determined due to rapid motion of the droplets) stems from silicate-carbonate melt immiscibility, as magmas enriched in alkaline carbonate and aluminosilicate components readily separate into silicate and carbonatite melts as they evolve (*52*). Thereafter, britholite that was contained in the melt droplets coalesced and gradually transformed into bastnäsite in the temperature range of 580° to 500°C (Fig. 7, p8), and the remaining melt crystallized into calcioburbankite at 460°C (Fig. 7, p9). With further reduction in temperature, cryolite (Cry; Na_3_AlF_6_) crystallized from the remaining fluid at ~400°C (Fig. 7, p10), indicating that sodium, aluminum, and fluoride in run 11 were oversaturated relative to silica, calcium, and REEs. SEM-EDS analysis of the end products showed that bastnäsite and calcioburbankite were the sole REE minerals, indicating that the hydrothermal fluid was depleted of REEs at temperatures below 450°C. Despite that the bulk composition in run 11 (i.e., potassium-free) differs from that of typical carbonatite complexes, which are enriched in sodium and potassium, with alkalis commonly crystalizing as nepheline rather than cancrinite (*2*, *36*, *44*, *50*), it is, nevertheless, clear that sufficient sodium precipitation in magmatic silicate minerals is crucial for the subsequent mineralization of REEs in fluorcarbonates.

## DISCUSSION

### REE mobility as controlled by sodium, carbonate, and silica

It has been widely believed that REE (fluor)carbonate minerals in carbonatites deposit from hydrothermal fluids exsolved from carbonatitic magmas, with F^−^, Cl^−^, and SO_4_^2−^ being regarded as the principle complexing ligands for REEs (*2*, *11*–*15*, *19*, *21*, *33*). However, results from high *P-T* experiments on hydrothermal fluids containing halogens and/or sulfate ions show that poor REE solubilities (typically few millimolal) (*17*, *22*, *53*–*55*) and low fluid-melt partition coefficients (0.02 to 0.15) for REEs (*27*) inhibit generation of concentrated ore-forming liquids with REE concentrations up to several weight percent (*30*, *34*, *39*). Whereas REEs have been shown to be highly mobile in concentrated sulfate liquids under hydrothermal conditions (*16*, *18*), the role of sulfate during REE mobilization in carbonatites is still questionable, as these experiments were conducted on isolated sulfate fluids without consideration of competition for REE complexation with the carbonate ion. The widespread occurrence of REEs as alkaline carbonate daughter minerals (e.g., burbankite, carbocernaite, and ancylite) in melt-fluid inclusions (*30*, *32*) and the appreciable REE mobilization in carbonates in the presence of alkalis under high *P-T* conditions (*7*) make sodium and carbonate ions obvious candidates for REE mobilization. Accordingly, the results of this study provide clear evidence that Na_2_CO_3_ brine-melts are exceptionally efficient at transporting and concentrating REEs, as evidenced from our experiments yielding REE concentrations greater than 8 wt % and REE/Na ratio approaching 0.10 at 700°C (Table 1 and Figs. 1D and 2D). Note that, in all runs (4 to 11, S1, and S2) containing bastnäsite in the starting assemblage, transformation of bastnäsite to carbocernaite was essential for the subsequent dissolution of REEs in the brine-melts (Figs. 2 and 4 to 7 and figs. S1 to S3). The vital importance of sodium and carbonate ions for REE mobilization was further confirmed in our additional REE dissolution experiments using bastnäsite and concentrated aqueous salt solutions (K_2_CO_3_, Na_2_SO_4_, NaCl, etc.), which showed that the bastnäsite-to-carbocernaite transformation and REE dissolution did not occur under high *P-T* conditions. With the addition of silica in runs 2 and 3, the increase in REE solubilities and REE/Na ratio with increasing temperature was suppressed by half due to the crystallization of REEs as britholite above 550°C (Fig. 1, C and D). Even so, the REE concentrations and REE/Na ratios in silica-bearing brine-melts were still comparable to those in the REE ore-forming liquids from carbonatites (*30*, *34*, *39*). Therefore, we conclude that REE mobilization in highly evolved carbonatitic brine-melts is controlled by interaction with sodium and carbonate ions and that REE enrichment to ore-forming levels can be achieved without additional complexing agents.

Examination of Raman spectra measured from high *P-T*, REE-rich Na_2_CO_3_ brine-melts (runs 1 to 7) shows an intense ν_1_ band (C-O symmetric stretching vibration) of CO_3_^2−^ and a much weaker ν_5_ band (C-O-H symmetric stretching vibration) of HCO_3_^−^ (Figs. 1B and 2B). Using the relative peak intensities and the Raman scattering coefficients for HCO_3_^−^ and CO_3_^2−^ in an aqueous liquid, the HCO_3_^−^/CO_3_^2−^ molar ratio was estimated to be <0.1 in the high *P-T* Na_2_CO_3_ brine-melts. This is lower than that in a low-density Na_2_CO_3_ hydrothermal fluid (e.g., >3.0) by an order of magnitude (*28*). Furthermore, molecular dynamics simulations show that REE(III)-CO_3_^2−^ complexing in aqueous fluids is orders of magnitude more stable than REE(III)-OH^−^ or REE(III)-HCO_3_^−^ complexing at high *P-T* conditions (e.g., 400°C and 0.4 kbar) (*37*). Therefore, despite the fact that our Raman spectra do not provide direct evidence for the speciation of REE complexes, it is reasonable to infer that the high REE mobility in concentrated Na_2_CO_3_ brine-melts is controlled by complexing with the carbonate ion without the hydroxyl ligand. The Na^+^ cation is well-known for its ability to provide charge balance in specific REE minerals (e.g., apatite) (*56*). Similarly, there may be a dynamic REE^3+^-Na^+^ coordination providing such charge balance in carbonatitic brine-melts. Our results contradict previous conclusions purporting that hydroxyl-carbonate polynuclear clusters are dominant transport species in dilute Na_2_CO_3_ hydrothermal fluids (*20*).

### REE deposition as carbonates and fluorcarbonates in carbonatites

On the basis of current models of mineral paragenesis and melt-fluid evolution in carbonatitic REE deposits, the mineralization of REEs is believed to be dominated by cooling of the ore-forming fluids (*11*, *13*, *14*) and/or their compositional alterations either resulting from interactions with the host rocks (*39*, *57*), mixing with low *P-T* meteoric water (*12*, *21*, *26*), or separation of volatiles, particularly CO_2_ (*24*, *31*, *38*, *50*). The results from our experiments provide clear evidence that deposition of REEs is controlled by cooling of the carbonate brine-melts. This is demonstrated by a rapid increase in REE solubilities above 500°C in the brine-melts during heating and REE mineral crystallization (bastnäsite, carbocernaite, or calcioburbankite) from the carbonate brine-melts upon cooling, via either continuous melt-fluid transition (Figs. 3, 4, 6, and 7 and figs. S1 and S3) or melt-fluid immiscibility (Fig. 5 and fig. S2). Depositional temperatures of REE minerals in our experiments (720° to 460°C; table S2) are 50° to 100°C lower than those from anhydrous carbonatite melts (*48*). Nevertheless, our crystallization temperatures are consistent with the presence of REE (fluor)carbonate minerals in carbonatitic pegmatites that were estimated to crystalize from carbonatite melts between 800° and 600°C (*2*, *29*, *31*, *46*, *58*). Conversely, REE solubilities in syn- and post-magmatic hydrothermal fluids (i.e., tens to hundreds of ppm) are orders of magnitude lower than in carbonate brine-melts, implying that the former are unable to transport REEs on large scales (*27*). Accordingly, hydrothermal processes such as mixing of magmatic hydrothermal fluids with meteoric water and separation of volatiles under relatively low *P-T* conditions (e.g., <400°C and 1 kbar) should not be considered as the principal depositional mechanism for the primary REE mineralization in carbonatites. We note that the crystallization temperatures for bastnäsite in our experiments (in the range of 500° to 580°C in Si-Al–bearing systems and >600°C in Si-Fe–bearing systems; table S2) are higher than the REE mineralization temperatures (e.g., 240° to 500°C) estimated by fluid inclusion microthermometry (*2*, *11*–*15*). This is most likely due to an underestimation of the trapping temperature for fluid-melt inclusions, on account of improper pressure calibration. Furthermore, as evidenced by widespread alteration of burbankite in hydrothermal fluids (*46*, *47*, *59*, *60*), REE carbonate minerals are commonly unstable and can be easily altered or overprinted by post-magmatic activity, leading to the formation of secondary bastnäsite, ancylite, parisite, or synchysite. These minerals appear to crystallize from hydrothermal fluids but are in situ alteration residuals, thus further complicating interpretation of the mineralization of REEs in carbonatites.

As demonstrated in this work, variations in carbonatitic melt compositions, particularly the alkali and silica concentrations, not only affect the capacity for REE mobilization in brine-melts but also determine the mineralization of REEs as either alkaline carbonates or fluorcarbonates (Figs. 3 to 7). Notably, fluoride has previously been believed to facilitate REE precipitation in carbonatitic melts and hydrothermal fluids (*12*, *17*, *19*): However, in our experiments, it was found to preferentially bond with calcium or sodium (Figs. 4 and 5 and figs. S1 and S2), rather than precipitating as REE fluorides or fluorcarbonates during cooling of the brine-melts. Consequently, the crystallization of REE fluorcarbonates (bastnäsite, parisite, synchysite, etc.), the predominant REE minerals that occur ubiquitously in carbonatites (*2*), requires not only the presence of fluoride but also the depletion of sodium in the carbonate brine-melts. The precipitation of aegirine (runs 9 and 10) or cancrinite (run 11) before crystallization of bastnäsite in our experiment (Figs. 6 and 7, fig. S3, and table S2), when taken in consideration with the well-established aegirine-augite-fluorite-baryte-bastnäsite mineral paragenesis in carbonatitic REE deposits (*2*, *31*, *38*, *44*), indicates that sufficient sodium precipitation in magmatic silicate minerals (aegirine, arfvedsonite, cancrinite, nepheline, etc.) is essential for reducing the sodium activity in carbonatitic melts or brine-melts and subsequent crystallization of REEs as fluorcarbonate minerals. Otherwise, as shown from runs 4 to 8, S1, and S2 in our experiment, while REE mineralization occurs from alkaline carbonate brine-melts containing little to no ferro- or aluminosilicate components, the presence of dissolved sodium results in REEs crystallizing as burbankite or carbocernaite. Despite occurring as primary REE minerals in some carbonatites (*46*, *47*, *61*), these minerals are far less stable than bastnäsite and commonly alter to secondary ancylite, bastnäsite, carbocernaite, and other gangue minerals by post-magmatic hydrothermal fluid alteration (*46*, *47*, *58*, *60*, *61*) and, consequently, attract relatively less attention during REE resource assessments.

## MATERIALS AND METHODS

### Sample preparation

Our high *P*-*T* experiments were carried out using a Bassett-type hydrothermal diamond anvil cell (HDAC; type V) (*62*, *63*), with samples being sealed in a gold (Au)–lined 400-μm-wide hole in the center of a 150- to 200-μm-thick rhenium (Re) gasket. REE minerals used in our experiments include bastnäsite crystals from Maoniuping REE deposit in Sichuan province, southwest China, and carbocernaite synthesized via reaction between bastnäsite or Ce_2_(CO_3_)_3_ (Kaimike Biochemical Co. Ltd., purity ≥ 99.9 wt %) and Na_2_CO_3_ reagents (Sinopharm Chemical Reagent Co. Ltd., purity ≥ 99.9 wt %), under hydrothermal conditions. Thirteen experimental runs with different starting materials were prepared under ambient *P-T* conditions (Table 1), which can be divided into four groups: (i) dissolution of carbocernaite in Na_2_CO_3_ brine-melts at the absence and presence of silica, for which carbocernaite was loaded into the HDAC together with Na_2_CO_3_, deionized water (run 1), and natural quartz chips (runs 2 and 3); (ii) dissolution of bastnäsite in Na_2_CO_3_ brine-melts (runs 4 to 7), where natural bastnäsite fragments were loaded together with Na_2_CO_3_ and deionized water; (iii) REE mineralization from alkaline carbonate brine-melts containing fluoride, calcium, silica, and iron or aluminum; in these runs, bastnäsite, Na_2_CO_3_ and H_2_O were loaded into the HDAC together with calcite (run 8) or with natural quartz, fluorite and hematite (runs 9 and 10) or corundum (run 11); and (iv) dissolution and crystallization of REEs during melt-fluid immiscibility, with bastnäsite and Na_2_CO_3_ (run S1) or bastnäsite, calcite, and Na_2_CO_3_ (run S2) loaded as the starting materials. By referring to the phase relations in the Na_2_CO_3_-H_2_O system (*28*), the liquid-vapor homogenization temperature was controlled to be <200°C for runs 1 to 11 and ~280°C for runs S1 and S2.

During the REE dissolution experiments (runs 1 to 7), each sample was heated at ~5°C/min, with Raman spectra of the solids (bastnäsite, calcite, or quartz) and liquids being measured at temperature intervals of 50° and 25°C below and above 500°C, respectively. Samples in runs 4 to 7 were held at 500°C for 1 to 3 hours, in order for the reaction between bastnäsite and Na_2_CO_3_ to proceed thoroughly, and subsequently heated to higher *P*-*T* conditions until all solids dissolved. The REE solubilities in runs 1 to 7 were calculated according to variations in the CO_3_^2−^ concentration, which was calibrated by using a Raman quantification method as described in our previous study (*28*). In the REE mineralization experiments (runs 8 to 11, S1, and S2), the samples were heated to high *P-T* conditions at 10°C/min until all solids dissolved or the temperature limit of the HDAC (850°C) was attained and then cooled down to room temperature at 5° to 10°C/min. Pressure of the samples in runs 1, 4 to 7, and S1 was estimated using isochoric *P-T* curves defined for the NaCl-H_2_O system (*28*, *64*), those in runs 2, 3, and 9 to 11 were estimated by extrapolating the *P-T* curves calibrated using the frequency shifts in the Raman peak of quartz at 464 cm^−1^ (*65*), and those in runs 8 and S2 were calibrated using the Raman peak of calcite at 1086 cm^−1^ (*66*).

### Raman spectral acquisition and data processing

The Raman spectra of mineral crystals (bastnäsite, calcite, fluorite, quartz, carbocernaite, burbankite, aegirine, britholite, cancrinite, cryolite, etc.) and CO_3_^2−^ (for concentration determination) in aqueous fluids or brine-melts under high *P*-*T* conditions were collected using a Renishaw inVia Raman micro-spectrometer, installed at the Institute of Mineral Resources, Chinese Academy of Geological Sciences, under excitation using a 532-nm neodymium-doped yttrium aluminium garnet laser (100 mW). The spectrometer is equipped with a Leica 10× long-working distance objective (numerical aperture of 0.25 and working distance of 17.7 mm), a 2400 g/mm grating, and a 1024 × 256 pixel charge-coupled device detector. The slit was set at 20 μm and the corresponding spectral resolution was 1.2 cm^−1^. During the experiments, the Raman spectra of the aqueous fluids or carbonate brine-melts were acquired with five accumulations of 10 s each in the spectral range of 100 to 4200 cm^−1^, with the focal position of the objective being fixed at ~20 μm below the interface between the liquid and the upper diamond anvil (*63*). The Raman spectra of the mineral crystals were acquired with three accumulations of 5 s each in the spectral range of 80 to 1200 cm^−1^, with peak positions being calibrated using the 546.07-nm emission line from a mercury (Hg)–bearing 5-W compact fluorescent lamp (*66*).

The intense ν_1_ band (C-O symmetric stretching vibration) of CO_3_^2−^ and the weak ν_5_ band (C-O-H symmetric stretching vibration) of HCO_3_^−^ were fitted using Gaussian + Lorentzian profiles, after baseline corrections using quadratic curves to remove the interference from the intense ν_1_ band of diamond. The O-H stretching vibration of water in the spectral range of 2800 to 3800 cm^−1^ was fitted using three Gaussian sub-bands, after baseline correction using a linear function (Figs. 1B and 2B) (*28*). Raman quantification for the CO_3_^2−^ concentration was carried out using the variation in the CO_3_^2−^/H_2_O Raman peak intensity ratio, which was calibrated using the same instrument that was used for measurements of K_2_CO_3_ solutions in our previous study (*28*). Errors in the Raman quantification results for CO_3_^2−^ concentration in the aqueous fluids or brine-melts were estimated to be within ±0.2 mol/kg.

### SEM-EDS analyses for REE mineralization products

The gold-lined Re gaskets along with the attached (along the inner rim) mineral crystals that formed upon completion of runs 3 to 11, S1, and S2 were collected carefully for SEM-EDS analyses. All samples were cleaned by adding two to three drops of deionized water to remove the Na_2_CO_3_ hydrates and other soluble salts precipitated on the surface, except for that from run 3, which was quenched by opening the sample chamber under 200°C and analyzed without sample cleaning. The Re gaskets along with mineral crystals in the sample chamber were mounted on an aluminum base and coated with gold. Analyses of the mineral morphology and element composition were conducted by using a Phenom XL benchtop SEM-EDS installed at the Civil and Resource Engineering School, University of Science and Technology Beijing. The accelerating potential was set at 15 kV, and the energy resolution of the EDS was 132 eV at the Mn Kα line.
