# Supplementary material for: Experimental evidence reveals the mobilization and mineralization processes of rare earth elements in carbonatites
Source: Sci Adv. 2024 Jul 3;10(27):eadm9118. doi: 10.1126/sciadv.adm9118 (PMC11221512; doi:10.1126/sciadv.adm9118)
Supplement: Supplementary file 1 — Supplementary Text Tables S1 and S2 Figs. S1 to S3 [file sciadv.adm9118_sm.pdf]

Science Advances
AAAS

Supplementary Materials for

Experimental evidence reveals the mobilization and mineralization processes of rare earth elements in carbonatites

Xueyin Yuan *et al.*

Corresponding author: Xueyin Yuan, xueyinyuan@live.com; Zhiming Yang, zm.yang@hotmail.com

Sci. Adv. **10**, eadm9118 (2024)
DOI: 10.1126/sciadv.adm9118

This PDF file includes:

Supplementary Text
Tables S1 and S2
Figs. S1 to S3

Supplementary text

RESULTS

Dissolution and crystallization of REE carbonate minerals during melt-fluid immiscibility

To investigate the mobilization and mineralization of REEs during melt-fluid immiscibility under high temperature (> 500 °C) but low pressure (< 4.5 kbar) conditions (*28*), two additional runs were prepared by loading bastnäsite with natrite (S1) or natrite and calcite (S2) into the HDAC, with vapor homogenization temperature being set at 278 and 286 °C, respectively. In contrast to the dissolution and crystallization of REE minerals during continuous melt-fluid evolution (Figs. 2, 4 and S1), the immiscibility between a carbonate melt and an aqueous fluid didn't preclude the dissolution of carbocernaite (formed from reaction between bastnäsite and Na_2CO_3 above 200 °C) above 500 °C, or the crystallization of REEs either as carbocernaite or calcioburbankite in the 650 – 480 °C temperature range (Figs. 5 and S2). However, the dissolution and crystallization of REE minerals were clearly limited within the carbonate melt phase, and the hydrothermal fluids equilibrated with the melts were dilute with very low CO_3^{2-} Raman peak intensities (Fig. 5). Given that the REE mobility is controlled by Na_2CO_3 enrichment in the aqueous liquids, it is clear that these syn-magmatic fluids contributed negligibly to REE mobilization and mineralization, which is consistent with the low REE partition coefficients between an aqueous fluid and a carbonate melt (*27*). SEM-EDS and Raman analyses showed that the mineralization products from run S1 (REEs in carbocernaite and fluoride in villiaumite, Fig. 5) were exactly the same as those from runs 4 – 7 (Fig. S1), and those from run S2 (REEs in calcioburbankite and nyerereite, fluoride in villiaumite, Fig. S2) were also highly similar to those from run 8 (Fig. 4). It is thus clear that the mineralization of REE carbonate minerals occurs during a late magmatic stage, and that the melt-fluid immiscibility has no influence on the REE mineralization products.

Tables

Table S1 Raman quantification results for the variation in CO_3^{2-} concentration during dissolution of Na_2CO_3 and REE carbonate minerals in runs 1 – 7

Temperature	Pressure [a]	Raman peak intensity			$c(CO_3^{2-})$	c(REE) [b]	REE/Na ratio
		CO_3^{2-}	H_2O	$R_{intensity}$			
**Run 1, carbocernaite + Na_2CO_3, vapor homogenized (Th_{L-V}) at 194 °C, Na_2CO_3 dissolved (Th_{NC}) at 398 °C, carbocernaite dissolved (Th_{REE}) at 642 °C**							
100 °C	1 bar	18809	20070	0.9372	4.21		
150 °C	5 bar	12574	17133	0.7339	3.38		
200 °C	0.02 kbar	10813	19303	0.5602	2.69		
250 °C	1.00 kbar	10709	17284	0.6196	3.17		
300 °C	1.82 kbar	12644	15729	0.8039	4.39		
350 °C	2.68 kbar	16237	16312	0.9954	5.77		
400 °C	3.50 kbar	22688	20154	1.1257	6.93		
425 °C	3.93 kbar	20073	18393	1.0914	6.96		
450 °C	4.32 kbar	18503	17337	1.0672	7.05		
475 °C	4.74 kbar	15237	15013	1.0149	6.99		
500 °C	5.16 kbar	14215	14511	0.9796	7.03	0.05	0.004
525 °C	5.54 kbar	14329	14660	0.9774	7.28	0.18	0.013
550 °C	5.97 kbar	14531	14589	0.9961	7.68	0.38	0.026
575 °C	6.36 kbar	13583	13402	1.0135	8.08	0.58	0.040
600 °C	6.78 kbar	13524	13041	1.0371	8.54	0.81	0.055
625 °C	7.18 kbar	13114	12083	1.0853	9.17	1.12	0.075
642 °C	7.50 kbar	13119	11722	1.1192	9.62	1.35	0.089
**Run 2, carbocernaite + Na_2CO_3 + quartz, Th_{L-V} at 187 °C, Th_{NC} at 365 °C, Th_{REE} not observed**							
365 °C	3.11 kbar	21240	20301	0.0552	6.18		
400 °C	3.68 kbar	18282	18961	0.0518	6.00		

450 °C	4.58 kbar	15944	18993	0.0486	5.71		
500 °C	5.47 kbar	14791	19207	0.0448	5.76	0.05	0.005
525 °C	5.94 kbar	15930	21169	0.0410	5.91	0.13	0.011
550 °C	6.36 kbar	15661	21166	0.0382	6.09	0.22	0.018
575 °C	6.76 kbar	15900	22043	0.0346	6.24	0.30	0.024
600 °C	7.17 kbar	13721	19543	0.0348	6.40	0.38	0.031
625 °C	7.58 kbar	13395	19761	0.0356	6.54	0.45	0.036
650 °C	7.96 kbar	13177	20110	0.0340	6.69	0.52	0.042
**Run 3, carbocernaite + Na_2CO_3 + quartz, Th_{L-V} at 178 °C, Th_{NC} at 336 °C, Th_{REE} not observed**							
350 °C	3.10 kbar	20322	20922	0.9713	5.64		
400 °C	4.00 kbar	20145	22422	0.8984	5.62		
450 °C	4.87 kbar	18364	23178	0.7923	5.43		
500 °C	5.80 kbar	16177	23934	0.6759	5.20	0.05	0.005
525 °C	6.22 kbar	14951	23040	0.6489	5.28	0.09	0.008
550 °C	6.63 kbar	10321	16327	0.6321	5.43	0.17	0.015
575 °C	7.01 kbar	10072	16044	0.6278	5.66	0.28	0.026
600 °C	7.46 kbar	10337	16904	0.6115	5.83	0.37	0.033
625 °C	7.85 kbar	10077	17398	0.5792	5.91	0.41	0.036
650 °C	8.24 kbar	11080	20335	0.5449	5.98	0.44	0.039
675 °C	8.65 kbar	10746	21232	0.5061	6.03	0.47	0.042
700 °C	9.04 kbar	9597	20800	0.4614	6.05	0.48	0.042
725 °C	9.49 kbar	8832	20694	0.4268	6.14	0.52	0.046
750 °C	9.88 kbar	8178	21426	0.3817	6.16	0.53	0.047
**Run 4, bastnäsite + Na_2CO_3, Th_{L-V} at 150 °C, Th_{NC} at 270 °C, Th_{REE} at 780 °C**							
100 °C	1 bar	27625	28885	0.9564	4.30		
200 °C	0.84 kbar	21207	24313	0.8723	4.24		
300 °C	2.69 kbar	21809	22848	0.9545	5.20		
350 °C	3.66 kbar	20331	22705	0.8954	5.22		
400 °C	4.60 kbar	18097	21993	0.8228	5.19		

450 °C	5.54 kbar	16141	24342	0.6631	4.68		
500 °C	6.52 kbar	15759	31979	0.4928	4.11		
550 °C	7.45 kbar	12776	25683	0.4974	4.60	0.25	0.027
575 °C	7.92 kbar	12470	26107	0.4776	4.73	0.31	0.034
600 °C	8.40 kbar	12928	26608	0.4859	5.04	0.47	0.050
625 °C	8.84 kbar	11883	23966	0.4958	5.38	0.64	0.068
650 °C	9.30 kbar	10955	22217	0.4931	5.65	0.77	0.081
675 °C	9.74 kbar	10355	22135	0.4678	5.78	0.84	0.087
700 °C	10.20 kbar	9698	22729	0.4267	5.82	0.86	0.089
725 °C	10.61 kbar	8836	23052	0.3833	5.85	0.87	0.090
750 °C	11.08 kbar	8140	23332	0.3489	5.94	0.92	0.095
780 °C	11.58 kbar	7172	24144	0.2971	5.99	0.94	0.097
**Run 5, bastnäsite + Na_2CO_3, $Th_{L\text{-}V}$ at 147 °C, Th_{NC} at 320 °C, Th_{REE} at 732 °C**							
100 °C	1 bar	19779	20311	0.9738	4.39		
200 °C	0.86 kbar	18837	20667	0.9115	4.43		
300 °C	1.80 kbar	22706	19393	1.1708	6.37		
320 °C	2.73 kbar	24475	20855	1.1736	6.53		
350 °C	3.70 kbar	22883	20443	1.1193	6.47		
400 °C	4.68 kbar	19512	19466	1.0024	6.22		
450 °C	5.65 kbar	16739	19774	0.8465	5.75		
500 °C	6.60 kbar	12563	17999	0.6980	5.33		
550 °C	7.54 kbar	14411	20913	0.6891	5.78	0.23	0.020
575 °C	8.00 kbar	12794	17690	0.7232	6.25	0.46	0.039
600 °C	8.47 kbar	14086	18140	0.7765	6.87	0.77	0.064
625 °C	8.89 kbar	14640	18568	0.7884	7.25	0.96	0.079
650 °C	9.36 kbar	13404	17142	0.7819	7.51	1.09	0.088
675 °C	9.83 kbar	13521	18069	0.7483	7.61	1.14	0.092
700 °C	10.25 kbar	13583	19146	0.7095	7.69	1.18	0.095
732 °C	10.86 kbar	11046	17172	0.6432	7.68	1.18	0.095

**Run 6, bastnäsite + Na_2CO_3, Th_{L-V} at 154 °C, Th_{NC} at 343 °C, Th_{REE} at 700 °C**							
100 °C	1 bar	27783	29718	0.9349	4.20		
200 °C	0.82 kbar	26491	30231	0.8763	4.26		
250 °C	1.73 kbar	25518	25348	1.0067	5.17		
300 °C	2.64 kbar	27976	24309	1.1508	6.26		
350 °C	3.60 kbar	33865	25311	1.3379	7.70		
400 °C	4.56 kbar	25655	20864	1.2296	7.53		
450 °C	5.53 kbar	22074	20201	1.0927	7.20		
500 °C	6.48 kbar	17683	20948	0.8442	6.21		
550 °C	7.35 kbar	18133	21699	0.8357	6.68	0.24	0.018
575 °C	7.84 kbar	20433	22949	0.8904	7.30	0.55	0.040
600 °C	8.31 kbar	19390	20848	0.9301	7.85	0.82	0.059
625 °C	8.76 kbar	18542	18975	0.9772	8.47	1.13	0.079
650 °C	9.22 kbar	17717	18471	0.9592	8.67	1.23	0.085
675 °C	9.67 kbar	15847	17043	0.9298	8.82	1.31	0.090
700 °C	10.12 kbar	14451	15928	0.9073	9.01	1.40	0.096
**Run 7, bastnäsite + Na_2CO_3, Th_{L-V} at 142 °C, Th_{NC} at 386 °C, Th_{REE} at 595 °C**							
150 °C	0.02 kbar	22386	26682	0.0587	3.89		
200 °C	1.07 kbar	21875	24120	0.0641	4.41		
250 °C	2.12 kbar	25134	24513	0.0513	5.27		
300 °C	3.11 kbar	29450	25122	0.0592	6.38		
350 °C	4.09 kbar	28246	21236	0.0714	7.66		
400 °C	5.07 kbar	30842	21022	0.0721	8.92		
450 °C	6.04 kbar	25660	19090	0.0686	8.71		
500 °C	7.01 kbar	23944	20812	0.0514	8.07		
550 °C	7.97 kbar	22583	19072	0.0372	8.86	0.40	0.023
575 °C	8.42 kbar	24311	19356	0.0446	9.63	0.78	0.045
590 °C	8.76 kbar	25186	19503	0.0443	10.05	0.99	0.056
600 °C	8.90 kbar	21002	16496	0.0499	10.06	1.00	0.057

Note: [a] – Pressure was estimated using the isochoric *P*-*T* curves defined for the NaCl-H_2O system (Runs 1, 4 – 7), or frequency shifts in the 464 cm^{-1} Raman peak of quartz (Runs 2 and 3);

[b] – REE concentration was calculated using the variation in CO_3^{2-} concentrations relative to that at 500 °C.

Table S2 Summary of the crystallization temperatures for REE and other minerals from carbonate brine-melts.

Minerals	800 °C	700 °C	600 °C	500 °C	400 °C	300 °C	200 °C
**Britholite**							
Fluorite							
Calcite							
Aegirine							
Cancrinite							
**Bastnäsite**							
**Carbocernaite**							
**Burbankite**							
Quartz							
Nyerereite							
Cryolite							
Villiaumite							
Natrite							

Figures

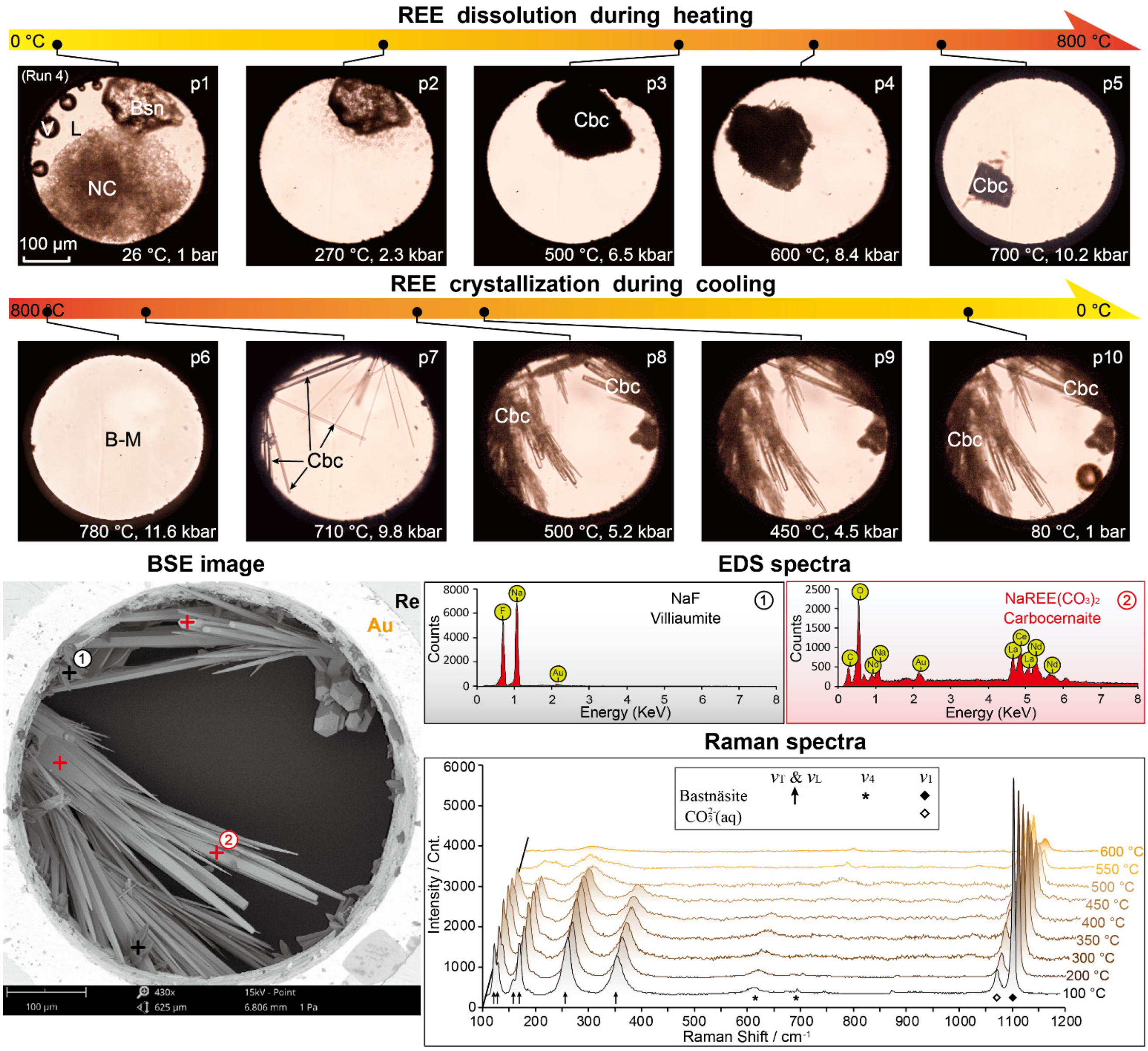

Figure S1 Dissolution and mineralization of REEs in hydrous Na_2CO_3 brine-melt (run 4). Photomicrographs p1 – p5 show the dissolution of bastnäsite (transforming into carbocernaite above 200 °C) in a hydrous Na_2CO_3 brine-melt; p6 – p10 show the crystallization of carbocernaite during cooling of the brine-melt. BSE image of the end product and EDS spectra of carbocernaite and villiaumite are shown for identification of the mineralization product. In-situ Raman spectra of bastnäsite show the transformation from bastnäsite into carbocernaite at 500 °C.

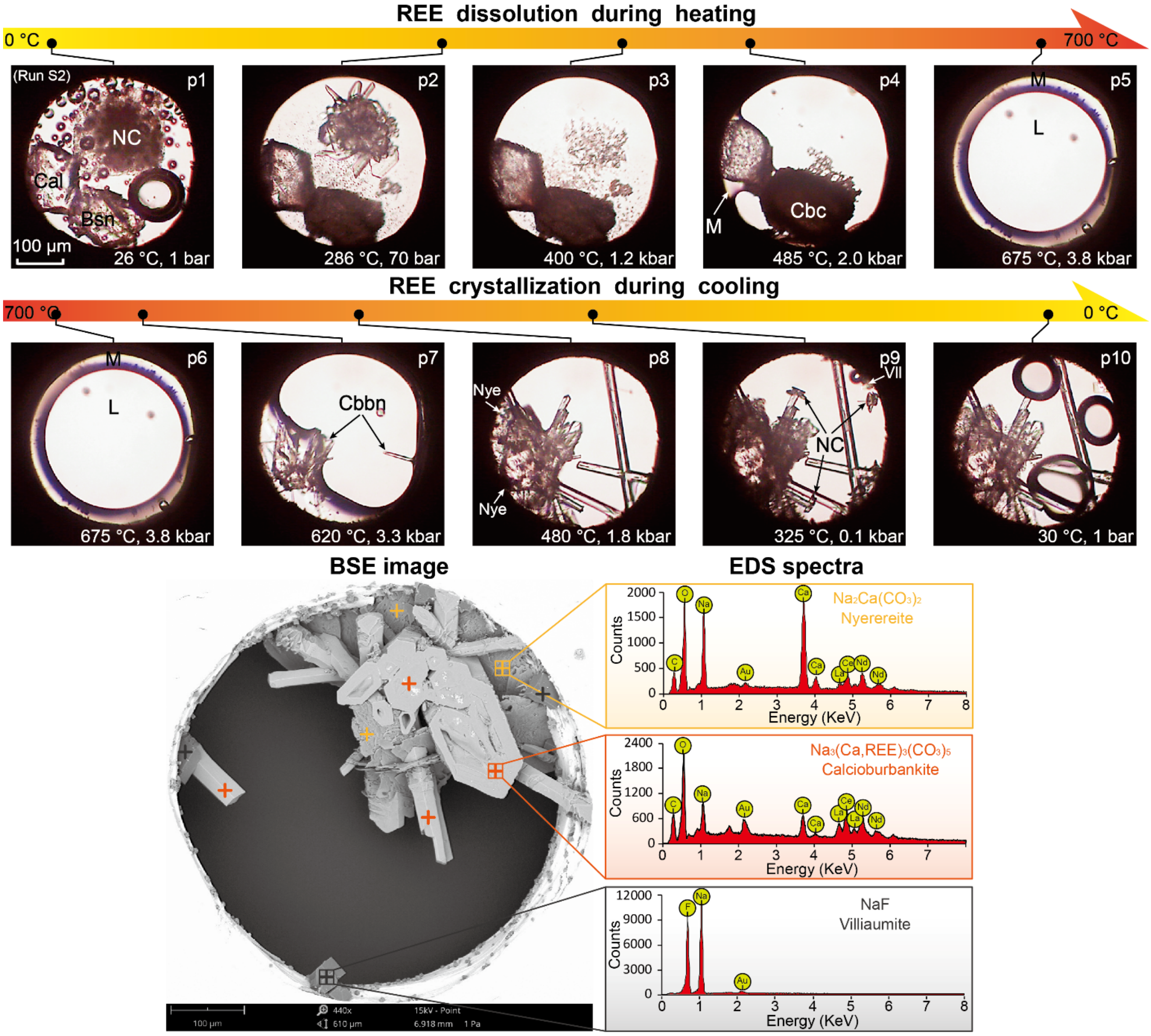

Figure S2 Dissolution and crystallization of REEs during melt-fluid immiscibility (run S2). Photomicrographs p1 – p5 show the melt-fluid immiscibility above 480 °C, and the dissolution of carbocernaite (formed from bastnäsite above 200 °C) and calcite into the melt; p6 – p10 show the precipitation of REEs in calcioburbankite and nyerereite from the melt, and fluoride as villiaumite from the hydrothermal fluid. BSE image of the product and EDS spectra of calcioburbankite, nyerereite and villiaumite are shown for identification of the mineralization product, where certain amounts of REEs were detected in the nyerereite crystals.

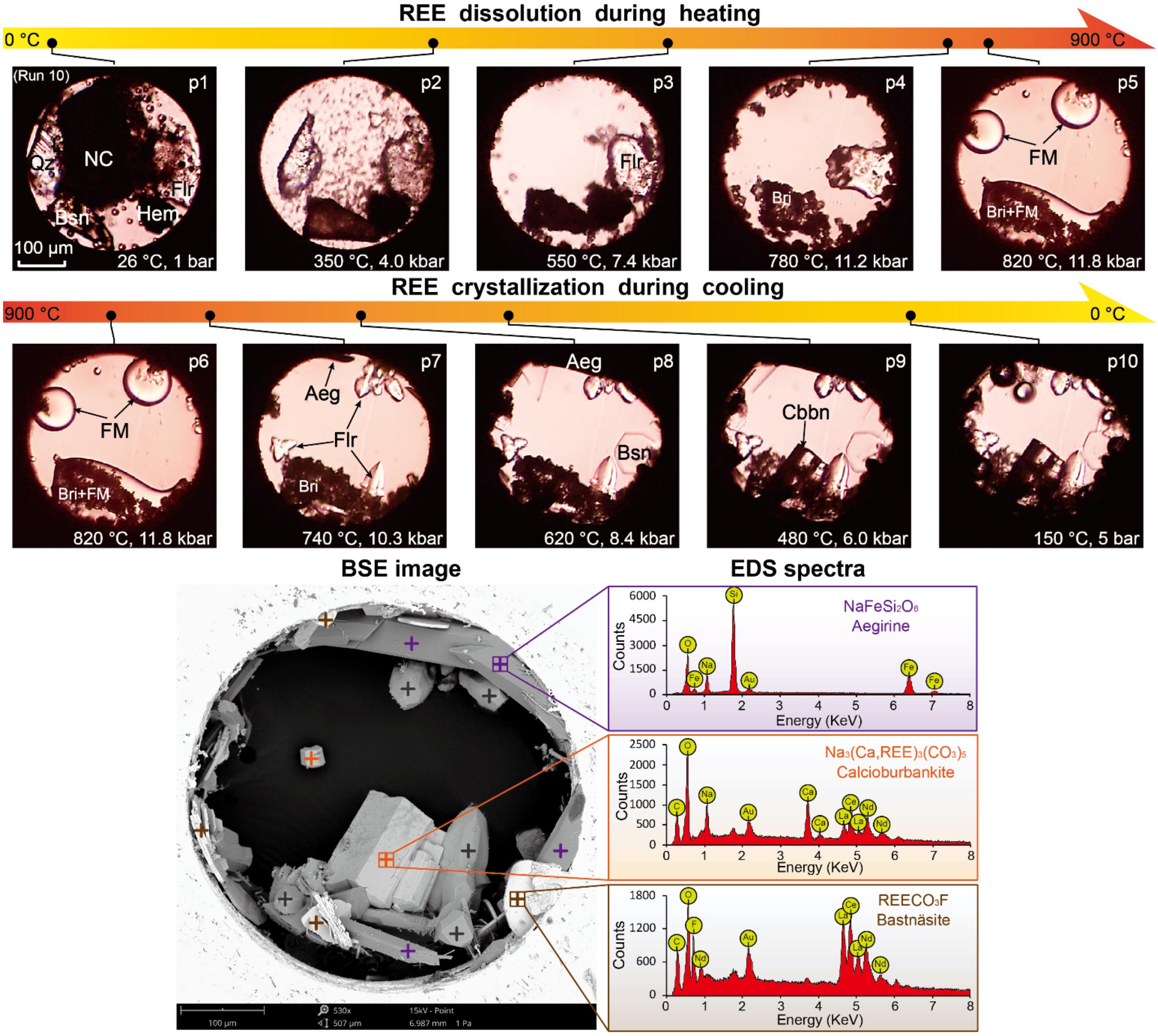

Figure S3 Dissolution and crystallization of REEs in a Si-Fe-bearing carbonate brine-melt (run 10). Photomicrographs p1 – p5 show the dissolution of natrite, bastnäsite, quartz and hematite into an alkaline carbonate brine-melt, and melting of fluorite into a fluoride melt above 820 °C; p6 – p10 show the crystallization of fluorite from fluoride melt, and sequential mineralization of aegirine, bastnäsite, and calcioburbankite along with the dissolution of britholite during cooling of the carbonate brine-melt. BSE image of the end product and representative EDS spectra of aegirine, calcioburbankite and bastnäsite are shown for identification of the mineralization products.
